# External Evaluation of Population Pharmacokinetic Models and Bayes-Based Dosing of Infliximab

**DOI:** 10.3390/pharmaceutics13081191

**Published:** 2021-08-03

**Authors:** Celine Konecki, Catherine Feliu, Yoann Cazaubon, Delphine Giusti, Marcelle Tonye-Libyh, Hedia Brixi, Guillaume Cadiot, Amélie Biron, Zoubir Djerada

**Affiliations:** 1 Department of Pharmacology, EA 3801, SFR CAP-Santé, Reims University Hospital, 51 rue Cognacq-Jay, CEDEX, 51095 Reims, France; celine.konecki@gmail.com (C.K.); catherine.feliu@univ-reims.fr (C.F.); 2 Institute Desbrest of Epidemiology and Public Health, INSERM, University Montpellier, 34000 Montpellier, France; yoann.cazaubon@chu-montpellier.fr; 3 Department of Pharmacology, Montpellier University Hospital, Avenue du Doyen Gaston Giraud, 34090 Montpellier, France; 4 Department of Immunology, Reims University Hospital, University of Reims Champagne-Ardenne, CEDEX, 51100 Reims, France; dgiusti@chu-reims.fr (D.G.); mtonyelibyh@chu-reims.fr (M.T.-L.); 5 Department of Hepato-Gastroenterology and Digestive Oncology, Robert Debré Hospital, Reims-Champagne-Ardennes University, CEDEX, 51100 Reims, France; hbrixi@chu-reims.fr (H.B.); gcadiot@chu-reims.fr (G.C.); abiron@chu-reims.fr (A.B.)

**Keywords:** infliximab, population pharmacokinetics, external evaluation, Crohn’s disease, ulcerative colitis, ankylosing spondylitis, psoriatic rheumatism, rheumatoid arthritis

## Abstract

Despite the well-demonstrated efficacy of infliximab in inflammatory diseases, treatment failure remains frequent. Dose adjustment using Bayesian methods has shown in silico its interest in achieving target plasma concentrations. However, most of the published models have not been fully validated in accordance with the recommendations. This study aimed to submit these models to an external evaluation and verify their predictive capabilities. Eight models were selected for external evaluation, carried out on an independent database (409 concentrations from 157 patients). Each model was evaluated based on the following parameters: goodness-of-fit (comparison of predictions to observations), residual error model (population weighted residuals (PWRES), individual weighted residuals (IWRES), and normalized prediction distribution errors (NPDE)), and predictive performances (prediction-corrected visual predictive checks (pcVPC) and Bayesian simulations). The performances observed during this external evaluation varied greatly from one model to another. The eight evaluated models showed a significant bias in population predictions (from −7.19 to 7.38 mg/L). Individual predictions showed acceptable bias and precision for six of the eight models (mean error of −0.74 to −0.29 mg/L and mean percent error of −16.6 to −0.4%). Analysis of NPDE and pcVPC confirmed these results and revealed a problem with the inclusion of several covariates (weight, concomitant immunomodulatory treatment, presence of anti-drug antibodies). This external evaluation showed satisfactory results for some models, notably models A and B, and highlighted several prospects for improving the pharmacokinetic models of infliximab for clinical-biological application.

## 1. Introduction

Infliximab (IFX) is a chimeric IgG1 monoclonal antibody against tumor necrosis factor alpha (TNFα) used in a wide range of inflammatory diseases [1], such as inflammatory bowel disease and spondyloarthropathies. Although its efficacy has been proven in several studies, therapeutic failure is still pretty common [2]. For example, in inflammatory bowel diseases, primary nonresponse occurs in almost one-third of patients, and secondary loss of response concerns 20 to 40% of patients initially responding [3]. Efficacy of IFX has been linked to serum concentrations in rheumatoid arthritis (RA) [4,5,6], ankylosing spondylitis (AS) [7], inflammatory bowel diseases (IBD) [8,9], and psoriatic patients [10]. Furthermore, suboptimal drug levels have been identified as one reason of therapeutic failure [2]. These observations have contributed to the use of therapeutic drug monitoring (TDM) for IFX, and therapeutic thresholds have been established in the main diseases: 3 mg/L for RA [11] and 5 mg/L for IBD [12].

Nevertheless, therapeutic optimization can be challenging due to IFX’s pharmacokinetics (PK), which is strongly influenced by patient-linked specificities [1,13,14,15]. Several TDM-based protocols have been tested to adapt IFX doses: stepwise dosing, proportional dosing, and model-based dosing [16]. An in silico assessment of these adaptive dosing strategies recently proved the superiority of the model-based approach using Bayesian forecasting based on a population PK model [16]. Mould et al. also assessed, in pediatric IBD patients, the benefit of Bayesian adaptive dosing, and showed that dashboard-driven dosing resulted in observed exposures generally contained within the range of exposures achieved with label dosing [17]. Even more recently, Strik et al. reported that the use of a Bayesian dashboard for IFX dosing in maintenance treatment for IBD patients reduced the incidence of loss of response and decreased fecal calprotectin levels compared to standard dosing [18]. Moreover, Santacana Juncosa et al. confirmed that IFX TDM combined with Bayesian forecasting improved the short-term clinical response in a real-world dataset of patients suffering from IBD [19].

However, many models have been published for IFX and choosing the best one for clinical practice can be difficult. Before using a model in clinical practice, an essential step after model building and qualification is model evaluation [20,21]. Yano et al. described model evaluation as follows: “the goal of model evaluation is objective assessment of the predictive ability of a model for domain-specific quantities of interest, or to determine whether the model deficiencies (the final model is never the ‘true model’) have a noticeable effect in substantive inferences” [22]. A model can be evaluated on the same population used for model building, i.e., internal evaluation, which can involve basic or more advanced methods, such as data splitting and/or the resampling technique, or on a different population coming from an external study, i.e., external evaluation [23,24,25,26,27,28]. A survey of the literature from 2002 to 2004 showed that external evaluation was conducted in only 7% of the published population PK models [29]. For IFX, only one external evaluation of one model has been published in the literature [30]. Thus, the present study aimed to evaluate published PK models of infliximab using a new dataset of patients treated at Reims University Hospital.

## 2. Materials and Methods

### 2.1. Literature Search

A systematic literature search for published population PK models of IFX up to February 2020 was performed on Pubmed, Google Scholar, Web of Science, Scopus, and summaries of gastroenterology, rheumatology, and pharmacology conferences. MeSH terms “Infliximab/pharmacokinetics” were chosen for the literature review, and the following keywords were combined: infliximab, pharmacology, population pharmacokinetics, to obtain the following search queries:

- (Infliximab [MeSH Terms]) AND (“1900” [Date-Publication]: “2020/02” [Date-Publication])
- (Infliximab/pharmacokinetics [MeSH Terms]) AND (“1900” [Date-Publication]: “2020/02” [Date-Publication])
- (Infliximab [MeSH Terms]) AND (Pharmacology [MeSH Terms]) OR (Pharmacology, Clinical [MeSH Terms]) AND (“1900” [Date-Publication]: “2020/02” [Date-Publication])
- (Infliximab) AND (“1900” [Date-Publication]: “2020/02” [Date-Publication]) AND (population) AND (pharmacokinetic)

A manual search for other relevant studies was conducted by inspecting the reference lists of identified journal articles. No restriction was made on pathology or study publication date. The study language was limited to English or French. The following information was extracted from the articles: model structure, typical population PK parameters, inter- and intra-individual variability, residual variability, covariates, software, estimation method, and internal evaluation procedure. Models were then selected for analysis if our dataset enabled their evaluation, i.e., if our population presented more than 20 patients resembling the construction population and all covariates were available.

### 2.2. External Evaluation Dataset

Data were collected retrospectively from the medical records of patients treated with IFX at Reims University Hospital from February 2016 to July 2020. This study was performed in accordance with the Declaration of Helsinki. As the study was based on medical data systematically recorded at Reims University Hospital for patient care, and authorized by the French national commission for data privacy (Commission Nationale Informatique et Libertés, CNIL MR004: 2206749v0), the study did not require approval by an ethics committee according to the French legislation on human research. Every patient received an information notice regarding the use of their data extracted during routine TDM procedures with their consent. No patient data in the external evaluation set had previously been included in the development of any of the models. Collected data included: IFX and anti-drug antibodies (ADA) concentration measurements, details about each infusion received (date of treatment initiation and of each infusion, dose administered, commercial drug name (Remicade^®^ or biosimilar), adverse effects), information on treated disease (date of diagnosis, co-medications, clinical and endoscopic evolution, disease activity scores), biological information (calprotectin, C reactive protein (CRP), albumin), and demographic data (sex, age, weight, smoking). IFX concentrations were measured using a validated enzyme-linked immunosorbent assay (Lisa Tracker, Theradiag, France), ranging from 0.3 mg/L to 16 mg/L. Data below the lower limit of quantification (LLOQ) were not included in the analysis. Data above the upper limit of quantification (ULOQ) were included if diluted and under 40 mg/L.

Data were included if at least one infusion of IFX and the respective concentration-time measurement were available. Data were excluded if there was any uncertainty about the time of dosing or drug concentration measurement and if dosing information was absent. Moreover, every evaluated study’s inclusion and exclusion criteria were respected so that every model was evaluated on a portion of the external evaluation population resembling its modelling population.

### 2.3. Evaluation of Predictive Performance

Each selected population PK model was separately implemented in Monolix (version 2019R2, Lixoft, Antony, France) [31] as described in the original article. Parameter values and covariate relationships for each model were set to those determined in the publications. Finally, predictions were generated using the doses, sampling times, and covariate values recorded in the evaluation data set.

Each model’s predictive performance was assessed graphically and by statistical analysis, as described by Nguyen et al. [20]. First of all, for each model, goodness-of-fit was assessed graphically by comparing observations to population predictions (PPRED) on the one hand and individual predictions (IPRED) on the other hand. The correlation coefficient (R²), mean error (ME), mean percentage error (MPE), and relative root mean square error (RMSE) were also calculated for PPRED and IPRED. Furthermore, residual error was evaluated on population-weighted residuals (PWRES), individual-weighted residuals (IWRES), and normalized prediction distribution errors (NPDE) [32]. PWRES and IWRES were plotted against PPRED and IPRED, respectively. NPDE represent a better approach than other metrics because they do not depend on a model approximation and have good statistical properties [32]. Since NPDE are assumed to follow a normal distribution (0;1), a quantile-quantile (QQ) plot and a representation of the distribution were produced. Several statistical tests were used as well: comparison of mean to 0 (Wilcoxon test), comparison of variance to 1 (Fisher test), Shapiro-Wilk normality test, and symmetry test. NDPE were also plotted against PPRED, time, and continuous covariates. Boxplots were used for categorical covariates as well as a Wilcoxon test for each situation (presence and absence of the covariate). Finally, each model’s prediction performances were evaluated using prediction-corrected visual predictive checks (pc-VPCs) performed by simulating 1000 data sets using each model. VPCs allow the comparison of the distribution of observations and predictions against an independent variable, time in this case.

Statistical analysis and graphs were performed using Rstudio (https://cran.r-project.org/ version 4.0.0, ggplot and ggformula packages, accessed on 8 May 2020) with data from Monolix. The only exceptions were pc-VPC and symmetry tests, which were conducted directly with Monolix. ME, MPE, and RMSE were calculated using the following formulas [21]:

$$\mathrm{ME}=\frac{1}{n}\times \overset{n}{\underset{i=1}{\sum }}\left(y_{i,pred}-y_{i,obs}\right)$$

$$\mathrm{MPE}\;\left(\%\right)=100\times \frac{1}{n}\times \overset{n}{\underset{i=1}{\sum }}\left(\frac{y_{i,obs}-y_{i,pred}}{y_{i,obs}}\right)$$

$$\mathrm{RMSE}\;\left(\%\right)=100\times \sqrt{\frac{1}{n}\times \overset{n}{\underset{i=1}{\sum }}\left(\frac{y_{i,obs}-y_{i,pred}}{y_{i,obs}}\right)²}$$

### 2.4. Bayesian Forecasting

The models were used to perform Bayesian dose simulations to assess their predictive capabilities. Each model’s population parameters were implemented in Monolix and fixed, and individual patient parameters were estimated using data of the *n*th infusion. These parameters were then used to simulate the concentrations obtained for these patients at infusions *n* + 1 and *n* + 2 using simulx (mlxR: R package version 4.0.6; Inria, Paris, France). The quality of the predictions was evaluated by calculating ME, MPE, and RMSE for the predictions of the concentrations obtained at infusions *n*, *n* + 1, and *n* + 2.

## 3. Results

### 3.1. Literature Search

**Figure 1:**
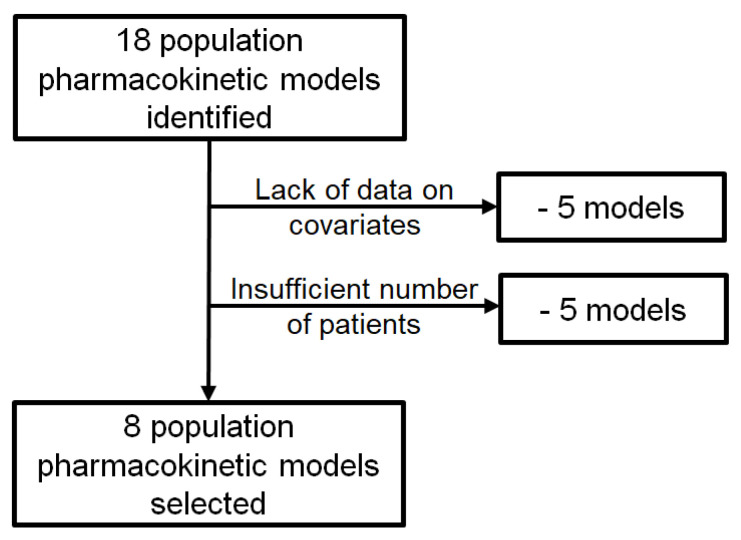
Flowchart of studies included in the evaluation.

A total of 18 population PK models for IFX were found: 5 one-compartment and 13 two-compartment models. Only eight of these models were selected for external evaluation [14,15,16,33,34,35,36] (Figure 1). The other 10 were excluded for lack of data: for five models, our dataset did not include essential covariates [13,37,38,39,40], such as FCGR3A genotype, body surface area (BSA), or previous anti-TFN exposure; and for the other five, our dataset did not have enough patients or concentration data compatible with the construction population [41,42,43,44,45]. Characteristics of these models are shown in Table 1 (evaluated models) and Supplementary Materials Table S1 (models not selected).

Model G and J were the only two models to take inter-occasion variability (IOV) into account, with an effect on the elimination clearance of 18.3% [36] and 3.43% [38], respectively. For model F, since the residual error model was not specified in the publication [15], a combined error model with the following parameters was implemented: σ_add_ = 0.80 mg/L and σ_prop_ = 22%. A combined model was chosen because it was used for 10 of the 18 published models, and the selected values reflect the median of these ten models’ parameters (see Table 1 and Supplementary Materials Table S1).

Applied covariates varied from one model to another, the most frequently used being weight (12 models out of 18, to which can be added two models considering BSA and one model considering fat free mass, both linked to weight), the presence of ADA (10 models out of 18), and sex (9 models out of 18). Serum albumin levels were frequently used in two-compartment models (7 models out of 13). Disease type was considered in only 3 models out of 18, but this can be explained by the fact that most of the remaining 15 models were developed on a population with a single disease (Crohn’s disease for three models, AS for two models, RA for two models, UC for two models) or a single group of diseases (IBD for five models).

### 3.2. External Evaluation Dataset

The external evaluation dataset consisted of 157 patients, including 82 women and 75 men. The main characteristics of the population are shown in Table 2. In total, 409 residual IFX concentrations were extracted. ADA were detected in 12 patients, with concentrations ranging from 20 to over 200 µg/L. Seven subpopulations were drawn from the primary database to match the populations on which the evaluated models were built. The characteristics of these subpopulations and those of the models being assessed are shown in Supplementary Materials Tables S2–S8.

### 3.3. Evaluation of Predictive Performance

#### 3.3.1. Goodness-of-Fit

**Figure 2:**
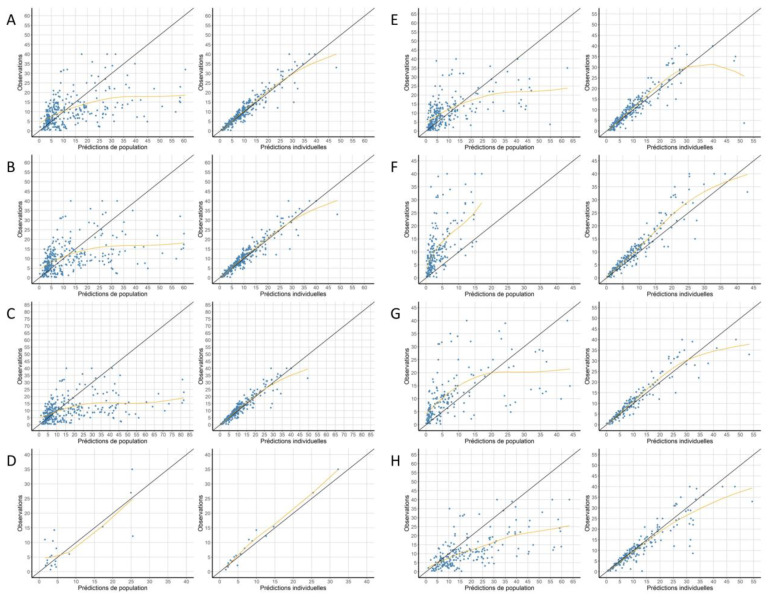
Graphs representing for each model (**A**–**H**) observations against population and individual predictions. The identity line is shown in black and a trend line (yellow) has been drawn for each model. Concentrations are shown in mg/L.

The results of the comparison of PPRED and IPRED to the observations for each model are shown in Figure 2 and Table 3. There was a relative dispersion of PPRED on either side of the identity line for most models: this shows imprecision, confirmed by R² capping at 45%. On this representation, numerous predictions were overestimated compared to the observations by models A, B, C, E, and H. Conversely, model F seemed to show a systematic negative bias, with most of the points lying above the identity line. For IPRED, the dispersion around the identity line was less important for most models, indicating a better accuracy of IPRED compared to PPRED. Additionally, no systematic bias was observed for IPRED for any model. This was confirmed by higher R², at least 85%, and a significant correlation (Spearman’s test, *p* < 0.001). The mean absolute error for IPRED was less than 1 mg/L for most models, except for F (−1.67 mg/L) and H (1.02 mg/L). The mean absolute error was not significantly different from zero for models A, B, C, and E (Student’s test, *p* < 0.05). Model H had the highest absolute values of ME, MPE (assessing accuracy), and RMSE (assessing precision) for PPRED. Models D, F, and G showed the best performance for IPRED in terms of MPE and RMSE but not in terms of ME.

#### 3.3.2. Residual Error

**Figure 3:**
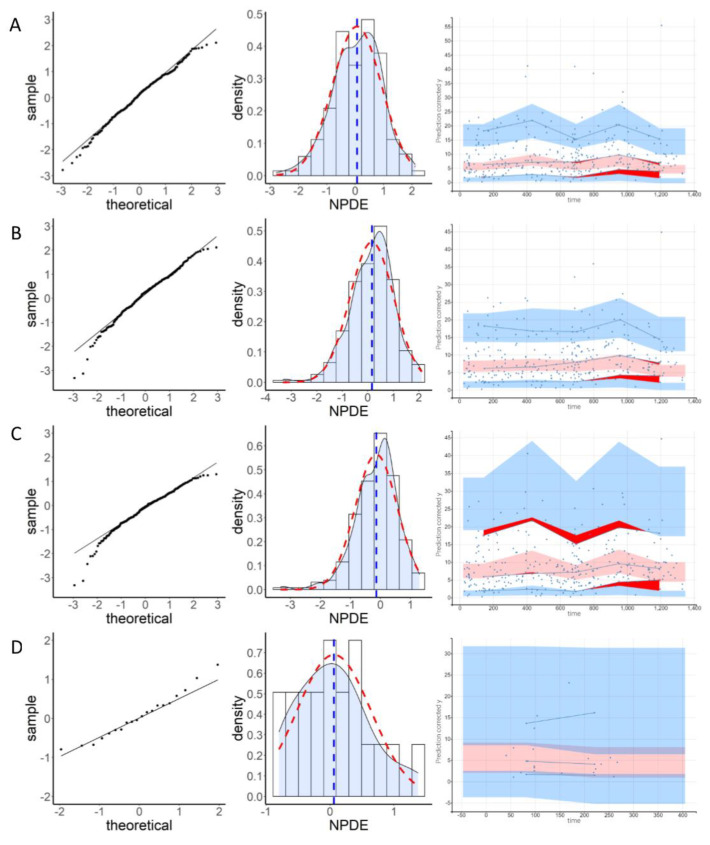

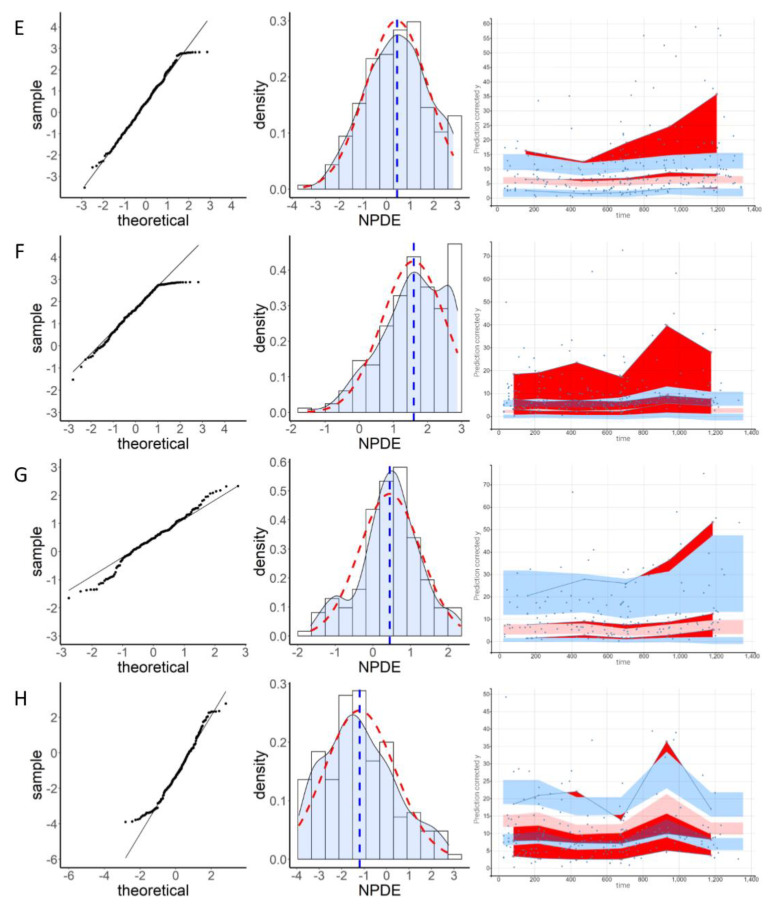
Distribution of NPDE and pcVPC for the 8 evaluated models (**A**–**H**). On the left, quantile-quantile graph of the NPDEs vs. theoretical normal distribution. In the middle, histogram and density of the NPDE distribution (blue dashed lines: mean; red dashed lines: density of the normal distribution). On the right, pcVPC (blue dots: observed concentrations; blue lines: 10th, 50th and 90th percentiles of observed concentrations; blue areas: prediction intervals of the 10th and 90th percentiles; light red area: prediction interval of the median; dark red area: outliers).

The results of the residual error assessment are summarized in Figure 3 and Table 4. Model D showed the best results for these tests, followed by model A. The assumption of normality of the NPDE distribution by the Shapiro-Wilk test was rejected for all models except models A and D. However, the variance of NPDE for model D was significantly different from 1 (2.963; *p* < 0.05). The mean NPDE was significantly positive for models B, E, F, and G (0.158, 0.441, 1.595, and 0.447, respectively, *p* ≤ 0.0001), highlighting an underestimation of concentrations. Conversely, a significantly negative mean of NPDE for models C and H (−0.140 and −1.199, respectively, *p* < 0.0001) pointed towards an overestimation of concentrations. The variance of the NPDE was significantly different from 1 for all models except F (Fisher’s test, *p* < 0.05), highlighting an overestimation of the variability for models A, B, C, D, and G (variance greater than 1), and an underestimation for models E and H (variance less than 1). The symmetry test was significant for models B and C (*p* < 0.05).

The results of the evaluation without covariates are presented in Supplementary Materials Figure S1 and Table S9. According to these, covariates’ addition improved the overall NPDE distribution for models A, B, C, and E. However, results were not notably different with or without covariates for models D, F, G, and H.

**Figure 4:**
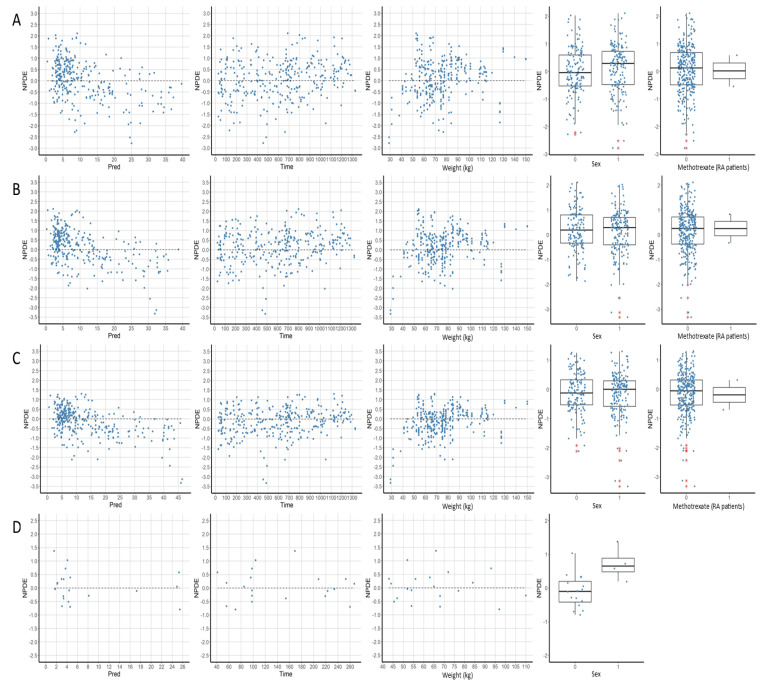

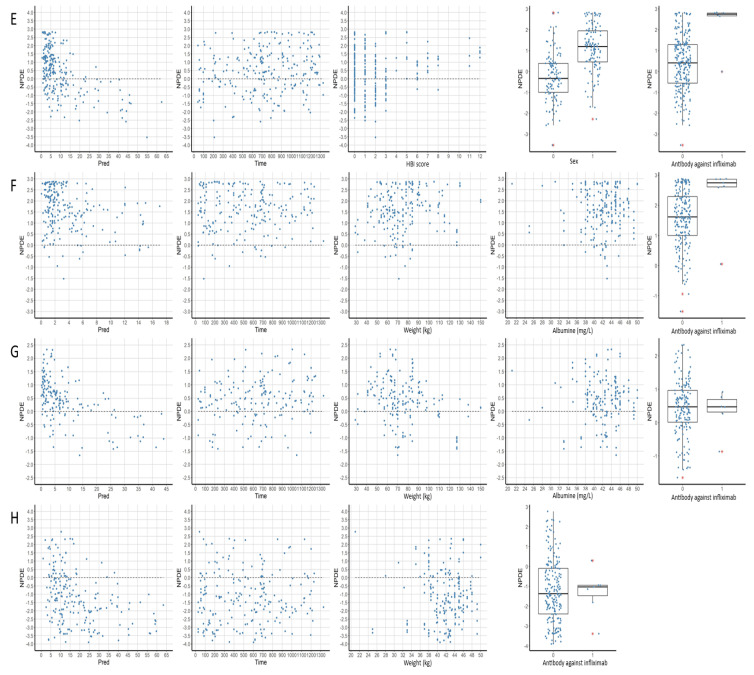
Evolution of NPDEs according to population predictions, time, and main covariates for the 8 evaluated models (**A**–**H**). Pred: population predictions, Sex: 0 = female and 1 = male except for model E.

Figure 4 and Supplementary Materials Figure S2 show the relationship between NPDE and PPRED, time, and continuous covariates considered in the models (weight, HBI score, and albumin), as well as a boxplot of NPDE for each condition of the categorical covariates (gender, pathology, concomitant treatment with another immunomodulator, presence of ADA, induction or maintenance period). The representation of NPDE against PPRED showed the same results as the graph of observations versus predictions and the statistical analysis of NPDE. Indeed, part of the predictions was overestimated by models A, B, C, E, and H. This manifested by systematically negative NPDE values for predictions exceeding a certain concentration (about 30 mg/L all models combined). The means of NPDE for these models were all negative, except for model E. Indeed, model E overestimated a part of the predictions but underestimated a greater number of them, which can be seen graphically. Model F underestimated the majority of the predictions. The same results were observed when studying the evolution of PWRES against PPRED (Supplementary Materials Figure S3). Conversely, on the representation of IWRES against IPRED, residuals were better distributed around the identity line, and less deviation was observed (Supplementary Materials Figure S3). Besides, no model showed a deviation of NPDE regarding time (Figure 4). On these representations, the points were fairly well distributed around the identity line for models A and B; there was a slight systematic bias for models C, E, and G; and a considerable bias for models F and H.

For continuous covariates, models A, B, and C overestimated concentrations in low weight patients (less than 40 kg, *p* < 0.05), and model E underestimated them in patients with high HBI scores (greater than 6, *p* < 0.0001). For categorical covariates, model G underestimated concentrations in patients not taking additional immunomodulatory therapy (*p* < 0.0001). Results for patients with ADA also appeared unsatisfactory for models E and F. However, the small number of patients (five) did not allow for a conclusion. The same was observed for gender in model D. The inclusion of covariates significantly improved pcVPC (Supplementary Materials Figure S1 and Figure 3), except for models F and G, where the improvement was slight. With covariates taken into account, the pcVPC of models A and B showed satisfactory results. Model C showed acceptable results as well, but the 90th percentile prediction range was very wide. All prediction intervals were also wide for model D, this being due to a lack of power. Model E showed an overestimation of the 90th percentile, which increased strongly with time. Model F showed an overestimation of the 10th, 50th, and 90th percentiles, overestimation for the 90th percentile being significant. Model G showed a slight overestimation of the 10th percentile and the median, and a larger overestimation of the 90th percentile, which had a considerable prediction interval. Model H showed an underestimation of the 10th percentile and the median, and variable results for the 90th percentile.

### 3.4. Bayesian Forecasting

Infusion simulations were performed for 14 patients with Crohn’s disease. Model H showed the best overall performance for the predictions of infusions *n*, *n* + 1, and *n* + 2 (Table 5). Model F showed the worst performances, with a critical negative bias (mean errors for infusions *n*, *n* + 1, and *n* + 2: −2.08 mg/L, −3.59 mg/L, and −6.58 mg/L, respectively). It was also observed that the results deteriorated from infusion to infusion: the predictions for the (*n* + 1)th infusion were less accurate and precise than for the *n*th infusion, and the same applied to those for the (*n* + 2)th infusion compared to the (*n* + 1)th infusion.

## 4. Discussion

Before considering using a PK model in current practice, evaluation is an essential step to assess accuracy, robustness, and predictive performances [46]. In addition to internal evaluation, which is necessary to test a model’s ability to describe the population with which it has been constructed, an external evaluation must be carried out [29]. This step evaluates not only the modelling procedure, but also other factors related to the study and the starting population. In this study, we evaluated eight PK models for IFX on a new independent population following published recommendations [20,23,32]. Performance varied from one model to another, and the evaluated models only partially satisfied the predefined criteria. Overall, all models showed a significant bias for PPRED, highlighting that these models will be of limited use for a priori dosage adjustments using only the population parameters and identified covariates. The mean error was much smaller for IPRED (less than 1 mg/L). However, the use of these models to perform Bayesian simulations showed that this error increased with the number of predicted infusions. The predictions of the (*n* + 1)th infusion were acceptable, so adaptive dosing using the individual parameters estimated by some of these models is possible. Nevertheless, determining infliximab concentrations will be necessary for each infusion to ensure that they remain within the therapeutic range.

Results for model A were the most satisfactory, followed closely by model B. Model D showed the best results when comparing observations to predictions and for residual error, but the prediction intervals for pc-VPC were very high. Indeed, model D was constructed considering only the first six months of infliximab treatment, and our database contained only 19 patients with these characteristics. The power of our external evaluation for this model was therefore limited. The results for models E, F, G, and H were the least satisfactory, particularly for the 90th percentile regarding pc-VPC. The study of residual errors for these models showed a systematic bias with underestimation of predictions for models E, F, and G, and overestimation for model H.

All authors had submitted their models to a partial internal evaluation during development. The procedure differed from one model to another: basic internal methods (assessment of the distribution of NPDE, PWRES, and IWRES) were used for models A, B, and C, whereas more advanced internal evaluation was performed for models E, F, and G (VPC and bootstrapping). Therefore, the recommendations concerning the evaluation of a population PK model have not been fully followed [20], which may explain the shortcomings observed in terms of prediction. Models B and C underwent advanced internal evaluation during their development since the starting population was divided into three groups: one group for model construction and two groups for performance evaluation. This explains the good predictive capabilities of model B observed in our study. The results of this evaluation were very close to those obtained here, especially on the bias observed graphically between the observations and the PPREDs, and on the accuracy and precision of the IPRED (bias 0.3 vs. −0.35 mg/L for model B; 0.1 vs. −0.29 mg/L for model C). Model C showed a better performance regarding the prediction of (*n* + 1)th infusions during the advanced internal evaluation compared to our external evaluation (bias for the (*n* + 1)th infusion: −0.4 vs. −1.17 mg/L). Model G is the only model to have been previously externally evaluated [30]. In agreement with our results, this evaluation showed a high correlation of observations and IPRED (R² = 97.6% vs. 92.3%) and a low relative bias (MIPE = −6.98% vs. MPE = −0.4%). Contrary to our evaluation, the NPDE mean and variance were close to the values expected for a normal distribution, i.e., 0 for the mean (0.01924, *p* > 0.05) and 1 for the variance (1.012, *p* > 0.05). The Shapiro-Wilk’s test, however, rejected the hypothesis of normality of the NPDE distribution (*p* < 0.05) as in our study.

The following factors can partially explain the different results from one model to another:

1. The population on which the model was built: Although an effort was made to ensure that each model was evaluated on a population that closely resembled the population on which it had been developed, not all patient characteristics were considered. Indeed, only pathology and the detection or not of ADA were used to select patients. Differences in other parameters, such as ethnicity, weight, albumin levels, disease severity, duration of infliximab treatment, or co-medication, may persist and partly explain the models’ non-applicability to our evaluation population. For example, model E was developed on a population with a median Harvey–Bradshaw index (score assessing the severity of Crohn’s disease) much higher than that of our population, showing a greater disease severity, which may influence the PK of infliximab [11,14].
2. The model construction study design: A mastered experimental design is necessary for a good estimation of the parameters. In PK studies, the crucial points to optimize are the number of subjects and samples as well as the sampling times, in order to minimize errors in the estimates [47]. Models A, B, C, E, and F have been developed using only residual concentrations, which may not be sufficient to estimate the distribution parameters correctly. However, the residual standard errors (RSE) of the population parameters were acceptable for all models except for the peripheral volume of model E (RSE(V_2_) = 32%). The RSE represents the estimate’s precision and must be under 30% for fixed parameters and under 50% for parameters of inter-individual variability [48].
3. The applied covariates: Many covariates influence the PK of infliximab, and those taken into account varied greatly from one model to another. Models A and B were the ones that considered the largest number of covariates and showed the best results in the external evaluation. However, how covariates are taken into account could be improved in some models. Indeed, if we take weight as an example, our results showed that the concentrations of patients with extreme weights were poorly predicted (*p* < 0.05). Moreover, the consideration of ADA by models E and F was also problematic (*p* < 0.0001). As shown by the internal evaluation results during model E’s development, the uncertainty of the relative effect of the antibody covariate on clearance was considerable (CI: 0.1–1.6). These results can be explained by the fact that none of the studies predicted the power required to estimate the covariates’ effects [49]. Indeed, the number of patients presenting these covariates in the construction populations was low (Supplementary Materials Tables S3, S5 and S6), and the imprecision on the parameters associated with the covariates was sometimes large (models B and C: RSE (β_MTX_) = 49% and 50%, respectively). Moreover, the small number of data or the presence of uninformative data predisposes to the phenomenon of shrinkage, thus explaining imprecise and potentially biased predictions [20]. This is particularly the case for model F, where shrinkage exceeded 40% for the estimation of all population parameters.

Our study is the first to have evaluated several IFX population PK models on a new population, but it had some limitations. The first one was its design: it was a monocentric and retrospective study, which limited the available data. Most of the concentration data collected for the evaluation consisted of residual concentration data, which do not allow a good appreciation of the distribution phase of infliximab and the identification of the peripheral compartment. It is, therefore, possible that one-compartment models may have had an advantage in the study design compared to two-compartment models. However, since this evaluation’s ultimate objective was to use these models in daily practice with only residual concentrations, it was essential to test them under these conditions. The second limitation of our study concerns the characteristics of the evaluation population. There is a significant imbalance in the pathologies represented in our population, with patients with Crohn’s disease being predominant. This disparity may influence the evaluation of models that include several pathologies, and prevented us from evaluating a number of models dealing with rheumatic pathologies, which are largely underrepresented in our population. Finally, the third limitation concerns the different measuring techniques used in the evaluated studies, which can bias the residual error model. Indeed, differences in concentration measuring techniques may lead to biased post hoc PK parameter estimates and decrease the predictability in Bayesian forecasting.

This study highlighted several perspectives for improving the PK models of infliximab, particularly concerning covariates. Some important covariates should be evaluated, particularly to consider patients presenting several pathologies. Indeed, in models A, B, and C, which used disease as a covariate, typical values of population parameters were associated with ankylosing spondylitis, while other pathologies were coded as categorical covariates. Patients with multiple pathologies were therefore not taken into account in these models. This concerns, for example, 22 patients in our starting population suffering from Crohn’s disease associated with ankylosing spondylitis. Most models using ADA as a covariate showed difficulties in predicting concentrations in these patients. Therefore, a specific model should be considered, as the PK of IFX is different in the presence of ADA [50]. Furthermore, given the central distribution of IFX, a different coding of the weight covariate would have to be tested, considering, for example, the ideal weight. Finally, since patients are followed over long periods, and certain covariates are linked to clinical response (weight and albumin in particular), it would be interesting to consider this additional variability. It would then be necessary to code these covariates into regressors or, at least, to assess IOV. IOV should make it possible to explain a significant part of the random variability and to improve the performance of Bayesian estimators [51,52]. Another perspective of improvement would be to try to build a new model using the parameters of the existing models. This could be done by aggregating the parameters of structurally identical models and create a meta-model. Another way would be to re-estimate and update dynamically the parameters of a given model using a new population. Finally, in order to compare the use of Bayesian methods for IFX dose adjustment to the usual strategy, and to obtain hard evidence of improved management, a prospective randomized controlled study is needed. Two studies have been performed very recently: a first prospective randomized but uncontrolled study using Fasanmade et al.’s model (model G) [19] and a second prospective randomized controlled study using Xu et al.’s model (model H) [18]. The first study showed an improvement in the percentage of patients in clinical remission from 65.7 to 80.4% (*p* < 0.0001), in the same group of patients, before and after using Bayesian methods. The second study showed the same improvement, with an increase from 64 to 88% of patients in remission (*p* < 0.017) when comparing the control group not receiving dose adjustments and the group of patients receiving Bayesian adjustments. However, models G and H showed less satisfactory results than others in our study, including models A and B. A new study could be initiated using one of these two models. These two studies also only involved IBD patients, so the use of Bayesian methods in other indications of infliximab remains to be evaluated.

## 5. Conclusions

In conclusion, this external evaluation showed satisfactory results for some models, notably models A, B, and C, and inferior results for others. Several paths for improvement were highlighted, particularly regarding covariates and the construction of a new model, as well as the realization of a new randomized controlled study.

## Figures and Tables

**Table 1:** Summary of the 8 models selected for the external evaluation.

Model	A	B	C	D
Reference	Passot et al., 2016	Ternant et al., 2017 PK	Ternant et al., 2017 TLD	Aubourg et al., 2015
Patients	218 patients with Crohn’s disease, UC, AS, PsA or RA, adults and children, no ADA detected	143 patients with Crohn’s disease, UC, AS, PsA or RA, adults and children, no ADA detected	143 patients with Crohn’s disease, UC, AS, PsA or RA, adults and children, no ADA detected	133 patients with Crohn’s disease, 6 first months of treatment, no ADA detected
Infliximab measurement	870 trough concentrations measured by ELISA (in-house)	Trough concentrations measured by ELISA (in-house)	Trough concentrations measured by ELISA (in-house)	Peak and trough concentrations measured by ELISA (in-house)
Tested covariates	Weight, sex, age (2 categories: pediatrics (<15) and adults (≥15)), disease (Crohn, UC, AS, PsA, RA), MTX treatment	Weight, sex, age (2 categories: pediatrics (<15) and adults (≥15)), disease (IBD, AS, PsA, RA), MTX treatment	Weight, sex, age (2 categories: pediatrics (<15) and adults (≥15)), disease (IBD, AS, PsA, RA), MTX treatment	Weight and sex
Clearance(s) (L/day)	CL = 0.23 × ${\left(\frac{WT}{67}\right)}^{0.603}\;$× e^0.181 × Sex^ × e^0.384 × Crohn^ × e^0.472 × UC^ × e^0.392^ ^× RA^ × e^−0.336^ ^× MTX^	CL = 0.24 × ${\left(\frac{WT}{67}\right)}^{0.52}\;$× e^0.36 × IBD^ × e^0.44 × RA^ × e^−0.37 × MTX^	CL = 0.20 × ${\left(\frac{WT}{67}\right)}^{0.68}\;$× e^0.43 × IBD^ × e^0.50 × RA^ × e^−0.47 × MTX^	CL = 0.336 × e^0.305^ ^× Sex^ Q = 1.992
Distribution volume(s) (L)	V = 5.2 × ${\left(\frac{WT}{67}\right)}^{0.277}\;\times$e^0.209 × Sex^ × e^0.399 × Crohn^ × e^0.417 × UC^ × e^−0.396 × Ped^	V = 5.3 × e^0.14 × Sex^ × e^0.25 × IBD^	V = 5.3 × e^0.19 × Sex^ × e^0.27 × IBD^	V_1_ = 2.6 × ${\left(\frac{WT}{60}\right)}^{0.22}\;\times$e^0.208^ ^× Sex^ V_2_ = 4.5
Residual error model	Combined error σ_add_ = 0.72 mg/L; σ_prop_ = 22.3%	Combined error σ_add_ = 0.80 mg/L; σ_prop_ = 22%	Proportional error σ_prop_ = 29%	Combined error σ_add_ = 2.3 mg/L; σ_prop_ = 21%
Software (algorithm)	Monolix (SAEM)	Monolix (SAEM)	Monolix (SAEM)	Monolix (SAEM)
Internal evaluation procedure	Goodness of fit plots, distribution of residuals plots	Learning (2/3) and validation (1/3) subsets, goodness of fit plots, distribution of residuals plots	Learning (2/3) and validation (1/3) subsets, goodness of fit plots, distribution of residuals plots	Not specified
**Model**	**E**	**F**	**G**	**H**
Reference	Buurman et al., 2015	Dotan et al., 2014	Fasanmade et al., 2011	Wojciechowski et al., 2017 Xu et al., 2012
Patients	42 patients with Crohn’s disease or UC	54 patients with Crohn’s disease or UC, adults only	692 patients with Crohn’s disease, children and adults	788 patients with Crohn’s disease, UC, RA or Kawasaki, children and adults
Infliximab measurement	188 trough concentrations measured by ELISA (in-house); ADA concentrations measured using a radioimmunoassay	169 trough concentrations measured and ADA concentrations measured by ELISA (Immunodiagnostik)	5757 peak and trough concentrations measured by ELISA	Not specified
Tested covariates	Clinical scores, duration and extension of disease, treatment period, weight, smoking, Crohn’s disease, UC, CRP, albumin, leucocytes, concomitant immunosuppressive drugs, prior infliximab use	Weight, sex, ADA, albumin	Age, weight, sex, race, AST, ALT, alkaline phosphatase, bilirubin, CRP, ALB, total protein, platelet and white blood cell counts, concurrent use of immunomodulators, ADA	Age, weight, albumin, disease type, ADA
Clearance(s) (L/day)	CL = 0.199 × 1.345^Sex^ × 1.722^ADA^ × 1.40^Period^ Q = 0.068	CL = 0.381 × ${\left(\frac{WT}{70}\right)}^{0.612}$ × ${\left(\frac{ALB}{40}\right)}^{-1.39}$ × (1 + 1.59 × ADA) Q = 0.122 × ${\left(\frac{WT}{70}\right)}^{1.15}$	CL = 0.3523 × ${\left(\frac{WT}{65}\right)}^{-0.313}\times {\left(\frac{ALB}{41}\right)}^{-0.855}\times$1.292^ADA^ × 0.863^IMM^ Q = 0.1469	CL = 0.294 × ${\left(\frac{WT}{70}\right)}^{0.614}\times {\left(\frac{ALB}{30}\right)}^{-1.17}\times$e^0.257^^×^^ADA^ Q = 0.079 × ${\left(\frac{WT}{70}\right)}^{1.1}$
Distribution volume(s) (L)	V_1_ = 4.94 × 0.964 × (HBI-6) V_2_ = 3.13	V_1_ = 2.37 × ${\left(\frac{WT}{70}\right)}^{0.696}$ V_2_ = 1.37 × ${\left(\frac{WT}{70}\right)}^{0.604}$	V_1_ = 3.406 × ${\left(\frac{WT}{65}\right)}^{-0.233}$ V_2_ = 1.274 × ${\left(\frac{WT}{65}\right)}^{-0.588}$	V_1_ = 3.33 × ${\left(\frac{WT}{70}\right)}^{0.691}$ V_2_ = 1.14 × ${\left(\frac{WT}{70}\right)}^{0.59}$
Residual error model	Combined error σ_add_ = 0.98 mg/L; σ_prop_ = 21.7%	Not specified	Combined error σ_add_ = 0.371 mg/L; σ_prop_ = 29.2%	Proportional error σ_prop_ = 17.5561%
Software (algorithm)	NONMEM (FOCE)	NONMEM	NONMEM	NONMEM
Internal evaluation procedure	Visual predictive checks, bootstrap analysis	Goodness of fit plots, visual predictive checks	Bootstrap analysis, condition number	Not specified

UC: ulcerative colitis, AS: ankylosing spondylitis, PsA: psoriatic arthritis, RA: rheumatoid arthritis, ELISA: enzyme-linked immunosorbent assay, MTX: methotrexate, WT: weight, IBD: inflammatory bowel disease, Ped: pediatrics, ADA: anti-drug antibodies, ALB: albumin levels, HBI: Harvey-Bradshaw index, IMM: concurrent use of immunomodulators, SAEM: stochastic approximation expectation-maximization, FOCE: first order conditional estimation.

**Table 2:** Baseline patients’ characteristics of the external evaluation dataset.

Characteristic	Median [Min–Max] or Number (%)
Female	82 (52.2)
Age (years)	39.5 (8.5–87.7)
Weight (kg)	68 (24–150)
Number of infusions per patient	23 (2–45)
Time between two infusions (days)	44 (14–79)
Administered dose per infusion (mg)	400 (180–1000)
Number of samples per patient	2 (1–6)
Infliximab concentration (mg/L)	8.68 (0–439)
Antibody against infliximab (patients)	12 (7.6)
Disease	
- Crohn’s disease	116 (73.9)
- Ankylosing spondylitis	22 (14.0)
- Ulcerative colitis	18 (11.5)
- Psoriatic arthritis	3 (1.9)
- Rheumatoid arthritis	3 (1.9)
Other immunomodulators	
- Azathioprine	49 (31.2)
- Methotrexate	31 (19.7)
- 6-mercaptopurin	6 (3.8)
- Other	2 (1.3)

**Table 3:** Comparison of population predictions (PPRED) and individual predictions (IPRED) to observations for the 8 evaluated models.

Model	Observations vs. PPRED					Observations vs. IPRED				
	R² (%)	ME (mg/L) [CI]	*p*	MPE (%)	RMSE (%)	R² (%)	ME (mg/L) [CI]	*p*	MPE (%)	RMSE (%)
A	34.6	0.04 [−0.81; 0.90]	0.9180	−58.4	279.0	90.3	−0.33 [−0.63; −0.02]	0.0343	−10.7	88.6
B	34.7	−0.59 [−1.48; 0.29]	0.1877	−41.9	236.0	90.5	−0.35 [−0.64; −0.05]	0.0212	−9.5	84.8
C	32.0	2.45 [1.40; 3.50]	<0.0001	−94.5	326.9	90.6	−0.29 [−0.60; 0.03]	0.0734	−9.0	89.9
D	27.7	−1.04 [−3.55; 1.46]	0.3940	−20.5	97.0	91.8	−0.74 [−1.45; −0.02]	0.0454	−5.1	43.4
E	33.7	−0.92 [−2.14; 0.31]	0.1413	−20.4	169.5	85.6	−0.45 [−1.02; 0.13]	0.1253	−16.6	115.0
F	41.3	−7.19 [−8.17; −6.21]	<0.0001	53.2	96.6	91.5	−1.67 [−2.12; −1.22]	<0.0001	8.6	43.7
G	42.5	−2.69 [−4.10; −1.28]	0.0002	3.5	145.0	92.3	−0.53 [−1.05; −0.01]	0.0442	−0.4	42.8
H	45.4	7.38 [5.88; 8.87]	<0.0001	−151.8	428.0	87.0	1.02 [0.47; 1.57]	0.0003	−39.6	299.8

R²: correlation coefficient, ME: mean error, CI: 95% confidence interval, *p*: Student’s test, MPE: mean percentage error, RMSE: root mean square error.

**Table 4:** Mean, variance, and results of normality and symmetry tests of the NDPE distribution for the 8 evaluated models.

Model	Mean	*p*	Variance	*p*	Normality (*p*)	Symmetry (*p*)
**A**	−0.062	0.0972	1.279	0.0100	0.0783	0.0917
**B**	0.158	0.0001	1.331	0.0026	0.0003	0.0044
**C**	−0.140	<0.0001	2.135	<0.0001	<0.0001	0.0023
**D**	0.062	0.7841	2.963	0.0067	0.7220	0.5446
**E**	0.441	<0.0001	0.576	<0.0001	0.0197	0.9934
**F**	1.595	<0.0001	1.092	0.4333	<0.0001	0.1131
**G**	0.447	<0.0001	1.402	0.0062	0.0431	0.5513
**H**	−1.199	<0.0001	0.404	<0.0001	0.0003	0.0514

Wilcoxon’s test for the comparison of the NPDE mean to 0, Fisher’s test for the comparison of the variance to 1, Shapiro-Wilk’s test for testing the hypothesis of normality.

**Table 5:** Simulation results of the *n*th, (*n* + 1)th, and (*n* + 2)th infusions for 7 models.

Model	Infusion *n*			Infusion *n* + 1			Infusion *n* + 2		
	ME (mg/L)	MPE (%)	RMSE (%)	ME (mg/L)	MPE (%)	RMSE (%)	ME (mg/L)	MPE (%)	RMSE (%)
A	−0.38	2.64	12.29	−1.35	14.98	23.38	−4.31	37.42	39.44
B	−0.50	4.71	14.07	−1.28	16.38	24.88	−4.52	40.60	41.96
C	−0.44	5.29	7.29	−1.33	16.23	25.10	−4.47	37.84	39.82
E	−0.93	3.54	48.75	−2.02	22.58	32.36	−4.78	47.62	50.73
F	−2.08	28.58	34.46	−3.59	37.23	42.67	−6.58	63.62	64.33
G	−1.02	13.37	14.22	−0.12	16.34	32.22	−4.45	40.43	43.56
H	0.57	−12.91	28.66	0.50	0.96	22.65	−2.83	26.58	32.52

ME = mean error, MPE = mean percentage error, RMSE = root mean square error. *N* = 14 patients for infusion *n* and *n* + 1, *N* = 4 patient for infusion *n* + 2.

## Data Availability

Due to ethical, legal or privacy concerns, and in accordance with the consent provided by participants, individual data cannot be shared.

## Supplementary Materials

The following are available online at https://www.mdpi.com/article/10.3390/pharmaceutics13081191/s1, Figure S1: Distribution of NPDE and pcVPC for the 8 evaluated models, covariates not taken into account, Figure S2: Boxplot of NPDE according to several categorical covariates for the 8 evaluated models; Figure S3: Evolution of residuals according to predictions: PWRES against PPRED and IWRES against IPRED, Table S1: Characteristics of models not selected for external evaluation, Table S2: Characteristics of the evaluation population and the construction population for model A, Table S3: Characteristics of the evaluation population and the construction population for model B and C, Table S4: Characteristics of the evaluation population and the construction population for model D, Table S5: Characteristics of the evaluation population and the construction population for model E, Table S6: Characteristics of the evaluation population and the construction population for model F, Table S7: Characteristics of the evaluation population and the construction population for model G, Table S8: Characteristics of the evaluation population and the construction population for model H, Table S9: Mean, variance and results of normality and symmetry tests of the NPDE distribution for the 8 evaluated models, covariates not taken into account.
