# Supplementary material for: External Evaluation of Population Pharmacokinetic Models and Bayes-Based Dosing of Infliximab
Source: Pharmaceutics. 2021 Aug 3;13(8):1191. doi: 10.3390/pharmaceutics13081191 (PMC8398005; doi:10.3390/pharmaceutics13081191)
Supplement: Supplementary file 1 [file pharmaceutics-13-01191-s001.zip › pharmaceutics-1312561-SM.pdf]

pharmaceutics

MDPI

Supplementary Materials: External Evaluation of Population Pharmacokinetic Models and Bayes-Based Dosing of Infliximab

Celine Konecki, Catherine Feliu, Yoann Cazaubon, Delphine Giusti, Marcelle Tonye-Libyh Hedia Brixi, Guillaume Cadiot, Amélie Biron and Zoubir Djerada

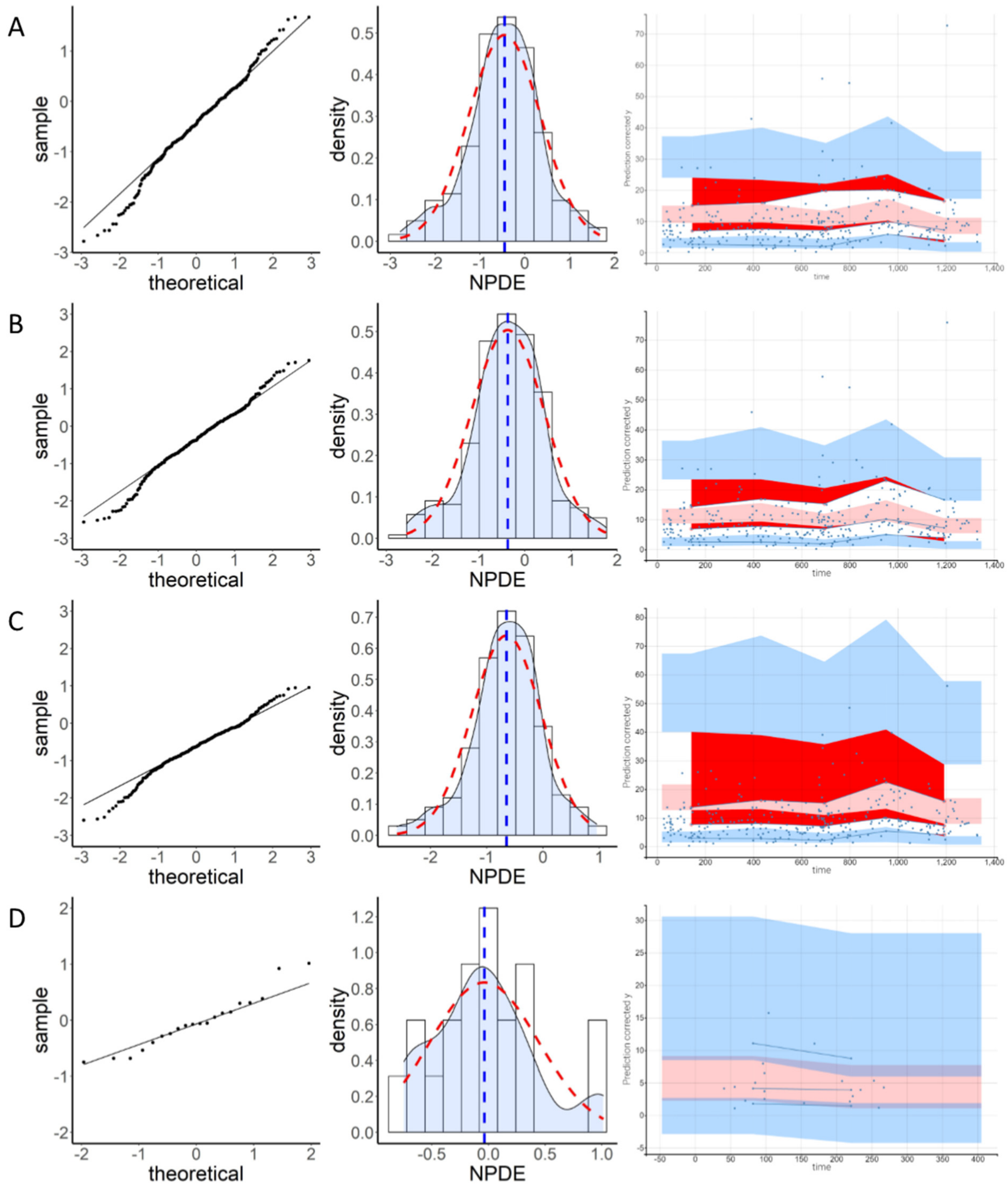

Figure S1 (continued)

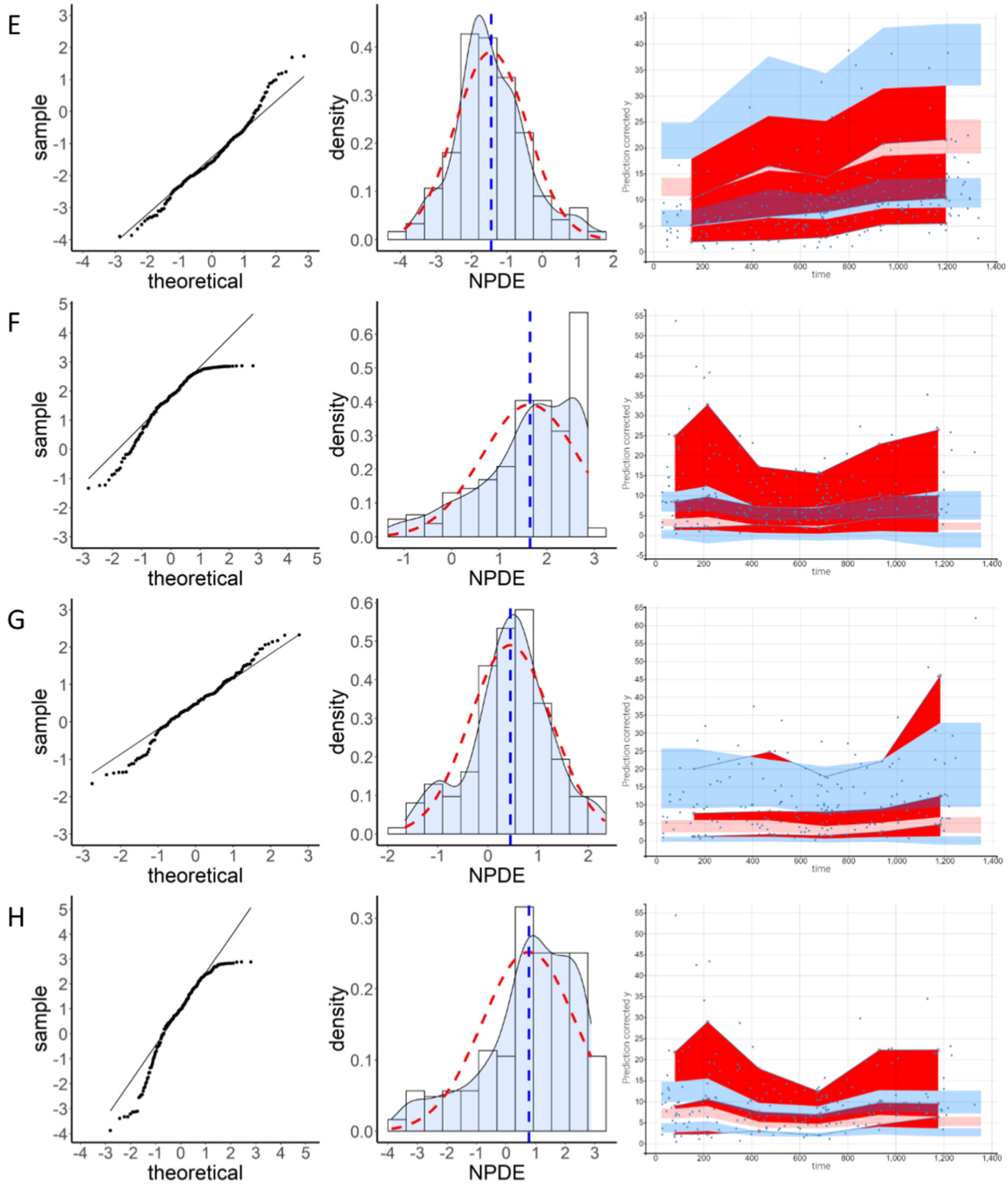

Figure S1. Distribution of NPDE and pcVPC for the 8 evaluated models, covariates not taken into account. On the left, quantile-quantile graph of the NPDEs vs. theoretical normal distribution. In the middle, histogram and density of the NPDE distribution (blue dashed lines: mean; red dashed lines: density of the normal distribution). On the right, pcVPC (blue dots: observed concentrations; blue lines: 10th, 50th and 90th percentiles of observed concentrations; blue areas: prediction intervals of the 10th and 90th percentiles; light red area: prediction interval of the median; dark red area: outliers).

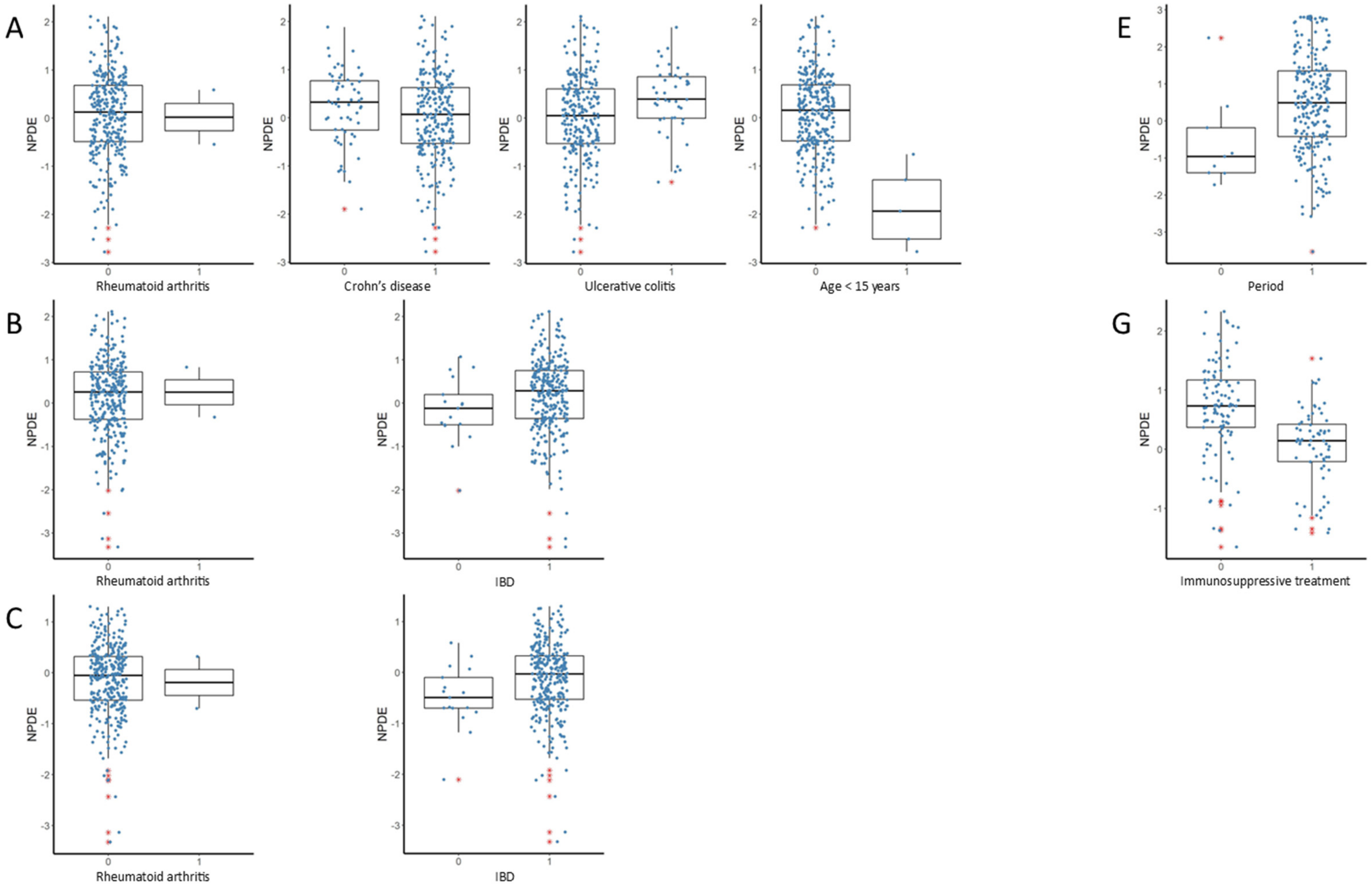

Figure S2. Boxplot of NPDEs according to several categorical covariates for the 8 evaluated models.

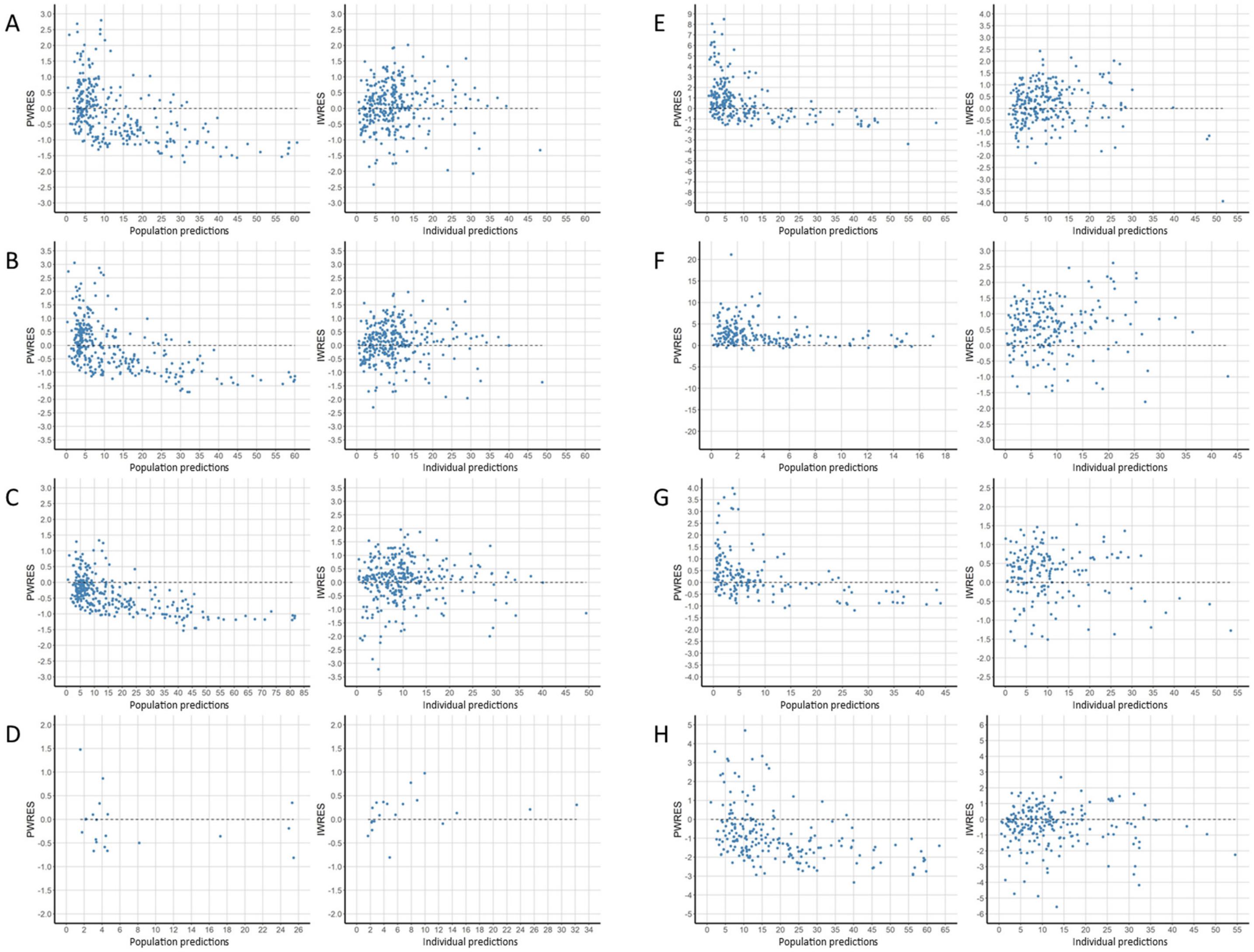

Figure S3. Evolution of residuals according to predictions: PWRES against PPRED and IWRES against IPRED. PWRES: Population Weighted Residuals, PPRED: population predictions, IWRES: Individual Weighted Residuals, IPRED: individual predictions

Table S1. Characteristics of models not selected for external evaluation.

Model	I	J	K
**Reference**	Fasanmade et al. 2009	Dreesen et al. 2018	Ternant et al. 2014a
**Patients**	728 patients with UC	204 patients with UC	111 patients with Crohn's disease
**Infliximab measurement**	4,310 peak and trough concentrations measured by ELISA	583 trough IFX and AAI concentrations measured by ELISA	546 concentrations measured by ELISA
**Tested covariates**	Age, WT, sex, race, renal function, liver function, CRP, ALB, total protein, hematology parameters, immunomodulator use, Mayo score, AAI	WT, FFM, sex, age, disease duration, concomitant CS and thiopurines at baseline, MES at baseline, pancolitis at baseline, time-varying CRP and ALB	Age, weight, hemoglobin level, WBC count, liver function, CRP, sex, FCGR3A genotype
**Clearance(s) (L/day) or elimination rate constant (/day)**	$CL = 0.407 \times \left(\frac{ALB}{41}\right)^{-1.54} \times 1.471AAI \times 0.764Sex$<br>Q = 7.14	$Ke = (0.0521 \times MES1 + 0.0543 \times MES2 + 0.0667 \times MES3) \times \left(\frac{ALB}{42}\right)^{-0.808} \times \left(\frac{CRP}{6.1}\right)^{0.0859}$	CL = 0.286 × e0.15 × FCGR3A x e0.055 × CRP
**Distribution volume(s) (L)**	$V1 = 3.29 \times \left(\frac{WT}{77}\right)^{0.538} \times 0.863Sex$<br>V2 = 4.13	$V = 6.34 \times 1.33 \times CS \times 1.23 \times PANC \times \left(\frac{FFM}{52}\right)^{0.544}$	V = 6.1
**Residual error model**	Combined error<br>σadd = 0.0413 mg/L ; σprop = 40.3%	Combined error<br>σadd = 0.09 mg/L ; σprop = 3.62%	Proportional error<br>σprop = 41%
**Software (algorithm)**	NONMEM (FOCE-I)	NONMEM	Monolix (SAEM)

Table S1 (continued)

Model	L	M	N
**Reference**	Brandse et al. 2017	Kevans et al. 2018	Ternant et al. 2008
**Patients**	332 patients with IBD	51 patients with IBD	33 patients with IBD
**Infliximab measurement**	997 concentrations measured by ELISA (Sanquin Laboratory), 81% residuals	IFX and AAI concentrations measured by the AnserTM IFX assay (Prometheus®)	478 trough and peak IFX concentrations measured by ELISA (in-house)
**Tested covariates**	Age, sex, length, weight, smoking, type, duration, location and behaviour of disease, immunomodulator use, selected laboratory values, previous anti-TNF exposure	WT, ALB, CRP, time from initiation of IFX therapy, AAI, concomitant immunomodulators	Age, WT, sex, disease, concomitant immunosuppressive treatment, AAI
**Clearance(s) (L/day)**	CL = $0.359 \times \left(\frac{WT}{72.3}\right)^{0.523} \times \left(\frac{ALB}{35.2}\right)^{-1.38}$ × e0.0521 × PE × e0.601 × AAI<br>Q = $0.0697 \times \left(\frac{WT}{72.3}\right)^{0.523}$	CL = $0.368 \times \left(\frac{WT}{61}\right)^{0.709} \times \left(\frac{ALB}{34}\right)^{-0.445}$ × -0.0373AAI<br>Q = $0.308 \times \left(\frac{WT}{61}\right)^{1.52}$	CL = 0.032 × AAI + 0.012 × (1-AAI)<br>Q=0.0054
**Distribution volume(s) (L)**	V1 = $4.72 \times \left(\frac{WT}{72.3}\right)^{0.473}$<br>V2 = $2.4 \times \left(\frac{WT}{72.3}\right)^{0.473}$	V1 = $3.33 \times \left(\frac{WT}{61}\right)^{0.64}$<br>V2 = $3.42 \times \left(\frac{WT}{61}\right)^{0.991}$	V1 = 2.3 × Sex + 1.1 × (1-Sex) + 1.7 × $\left(\frac{WT}{67}\right)$<br>V2 = 1.9
**Residual error model**	Not specified	Proportional error<br>σprop = 28.5%	Additive error<br>σadd = 1.04 mg/L
**Software (algorithm)**	NONMEM	NONMEM (FOCE)	Winnonmix (conditional first-order method)

Table S1 (continued)

Model	O	P	Q	R
**Reference**	Xu et al. 2008	Ternant et al. 2012	Palaparthy et al. 2019	Ternant et al. 2014b
**Patients**	247 patients with ankylosing spondylitis	26 patients with ankylosing spondylitis	649 patients with rheumatoid arthritis	84 patients with rheumatoid arthritis
**Infliximab measurement**	8137 peak and trough concentrations, measured by ELISA	507 peak and trough concentrations, measured by ELISA	4881 concentrations, measured by ELISA	412 concentrations, measured by ELISA
**Tested covariates**	Sex, BSA, disease activity, renal function, WBC, concomittant treatments	Sex, methotrexate, age, height, weight, BSA	Age, BSA, weight, sex, race, ethnicity, albumin, CRP, rheumatoid factor, WBC count, antibodies against infliximab, previous biologic use methotrexate dose	Sex, association with MTX and/or corticosteroids, age, weight, height, disease duration, infliximab treatment duration, CRP, disease activity score
**Clearance(s) (L/day)**	CL = (0.273 + (GB - 7.9) × 0.0106) × (1 + RF × 0.419)<br>Q = 3.06	CL = 0.21<br>Q = 2.3	CL = 0.015 × AAI0.478 × e0.255 × Sex<br>Q = 0.2	CL = 0.019 × e-0.36 × MTX × $\left(\frac{CRP}{3.2}\right)^{0.12}$<br>Q = 0.18
**Distribution volume(s) (L)**	V1 = 3.06 × $\left(\frac{BSA}{1.94}\right)^{0.744}$ × (1 - sex × 0.100)<br>V2 = 2.94	V1 = 2.3 × $\left(\frac{BSA}{1.89}\right)^{0.88}$<br>V2 = 1.8 × $\left(\frac{BSA}{1.89}\right)^{1.9}$	V1 = 3.57 × $\left(\frac{WT}{70}\right)^{0.669}$<br>V2 = 1.65	V1 = 2.3 × $\left(\frac{WT}{65}\right)^{1.2}$<br>V2 = 3.6
**Residual error model**	Combined error<br>σadd = 2.89 mg/L ; σprop = 22.9%	Combined error<br>σadd = 2.6 mg/L ; σprop = 18%	Proportional error<br>σprop = 77.0%	Combined error<br>σadd = 0.15 mg/L ; σprop = 16%
**Software (algorithm)**	NONMEM (FOCE)	MONOLIX (SAEM)	NONMEM	MONOLIX (SAEM)

UC: ulcerative colitis, IBD: inflammatory bowel disease, IFX: infliximab, ELISA: enzyme-linked immunosorbent assay, CRP: C-reactive protein, ALB: albumin, AAI: antibody against infliximab, WT: weight, PE: previous anti-TNF exposure, WBC: white blood cells, RF: renal failure, BSA: body surface area, FFM: fat free mass, CS: corticosteroids, MES: Mayo endoscopic subscore, BMI: body mass index, PANC: pancolitis.

Table S2. Characteristics of the evaluation population and the construction population for model A

Characteristic	Evaluation population	Construction population
Number of patients	135	218
Number of infliximab samples	303	870
Females	66 (49%)	141 (65%)
> 15 years old	133 (99%)	207 (95%)
Weight (kg)	70 [29-150]	67 [28.2-125]
Disease		
- Crohn's disease	101 (75%)	63 (29%)
- Ankylosing spondylitis or psoriatic arthritis	12 (9%)	121 (56%)
- Ulcerative colitis	20 (15%)	16 (7%)
- Rheumatoid arthritis	2 (1%)	18 (8%)

Table S3. Characteristics of the evaluation population and the construction population for models B and C

Characteristic	Evaluation population	Construction population
Number of patients	140	143
Number of infliximab samples	310	Not specified
Females	66 (47%)	49 (34%)
Weight (kg)	72.5 [29-150]	67 [56-80]
Disease		
- Crohn's disease or ulcerative colitis	126 (90%)	50 (35%)
- Ankylosing spondylitis or psoriatic arthritis	12 (9%)	80 (56%)
- Rheumatoid arthritis	2 (1%)	13 (9%)
Methotrexate use among patients with rheumatoid arthritis	21 (15%)	8 (1%)

Table S4. Characteristics of the evaluation population and the construction population for model D

Characteristic	Evaluation population	Construction population
Number of patients	19	133
Number of infliximab samples	20	Not specified
Females	15 (79%)	79 (59%)
Weight (kg)	65 [43-110]	60 [41-120]

Table S5. Characteristics of the evaluation population and the construction population for model E

Characteristic	Evaluation population	Construction population
Number of patients	95	42
Number of infliximab samples	238	188
Females	52 (55%)	22 (52%)
Harvey Bradshaw Index	1 [0-12]	6 [3-24]
Antibodies against infliximab	2 (2%)	2 (5%)

Table S6. Characteristics of the evaluation population and the construction population for model F

Characteristic	Evaluation population	Construction population
Number of patients	101	54
Number of infliximab samples	206	169
Weight (kg)	71 [29-150]	70 [40-112]
Albumin (mg/L)	42.5 [21-50]	40 [21-50]
Antibodies against infliximab	5 (5%)	17 (31%)

Table S7. Characteristics of the evaluation population and the construction population for model G

Characteristic	Evaluation population	Construction population
Number of patients	90	692
Number of infliximab samples	176	5757
Weight (kg)	68.5 [29-150]	64.5 [20.4-157.3]
Albumin (mg/L)	42.5 [21-50]	41 [20-55]
Antibodies against infliximab	5 (6%)	67 (9.7%)

Table S8. Characteristics of the evaluation population and the construction population for model H

Characteristic	Evaluation population	Construction population
Number of patients	101	788
Number of infliximab samples	206	Not specified
Weight (kg)	71 [29-150]	70
Albumin (mg/L)	42.5 [21 -50]	30
Antibodies against infliximab	5 (5%)	Not specified

Table S9. Mean, variance and results of normality and symmetry tests of the NDPE distribution for the 8 evaluated models, covariates not taken into account.

Model	Mean	p	Variance	p	Normality (p)	Symmetry (p)
**A**	−0.453	<0.0001	1.371	0.0010	0.0179	0.9380
**B**	−0.369	<0.0001	1.660	<0.0001	0.0085	0.9811
**C**	−0.649	<0.0001	2.502	<0.0001	0.0012	0.3273
**D**	−0.038	0.5706	4.149	0.0006	0.3287	0.6925
**E**	−1.449	<0.0001	0.901	0.2923	0.0018	0.0135
**F**	1.646	<0.0001	0.987	0.8851	<0.0001	0.0005
**G**	0.447	<0.0001	1.551	0.0004	0.0431	0.1902
**H**	0.772	<0.0001	0.426	<0.0001	<0.0001	0.0156

Wilcoxon's test for the comparison of the NPDE mean to 0, Fisher's test for the comparison of the variance to 1, Shapiro-Wilk's test for testing the hypothesis of normality.
